# Nutrition and Uterine Fibroids: Clinical Impact and Emerging Therapeutic Perspectives

**DOI:** 10.3390/jcm14207140

**Published:** 2025-10-10

**Authors:** Francesco G. Martire, Eugenia Costantini, Ilaria Ianes, Claudia d’Abate, Maria De Bonis, Giovanni Capria, Emilio Piccione, Angela Andreoli

**Affiliations:** 1 Department of Molecular and Developmental Medicine, Obstetrics and Gynecological Clinic, University of Siena, 53100 Siena, Italy; francescogmartire@libero.it (F.G.M.); eugenia.costantini22@gmail.com (E.C.); ilaria.ianes@gmail.com (I.I.); claudiadabate94@gmail.com (C.d.); mariadebonis@gmail.com (M.D.B.); 2 SSD Centre for Artificial Nutrition, Clinical and Home Care, Antonio Cardarelli Regional Hospital, 86100 Campobasso, Italy; giovannicapria@yahoo.com; 3 Gynecology and Obstetrics, Department of Surgical Sciences, University of Rome “Tor Vergata”, 00133 Rome, Italy; 4 Residency Program of Gynecology and Obstetrics, Catholic University “Our Lady of Good Counsel”, 1000 Tirane, Albania; 5 Residency Clinical Program of “Nutritional Sciences”, Catholic University “Our Lady of Good Counsel”, 1000 Tirane, Albania; angela.andreoli@uniroma2.it

**Keywords:** estrogens, fibroids, nutritional factor, symptoms, therapy

## Abstract

Nutritional factors play a crucial role in many gynecological disorders, particularly those influenced by estrogen. Uterine fibroids are benign tumors that affect a large proportion of women of reproductive age, especially between 30 and 40 years. These lesions may cause significant symptoms, including pelvic pain, heavy menstrual bleeding, and infertility. In younger women, the onset of fibroids is often associated with familial and genetic predisposition, whereas in adulthood, hormonal influences linked to environmental factors and states of exogenous or endogenous hyperestrogenism are more frequently observed. In both contexts, supportive management through an appropriate diet may provide clinical benefit. Although the precise pathogenesis remains incompletely understood, hormonal, genetic, and environmental components—particularly hyperestrogenism—are considered key contributors to fibroid development. Current evidence suggests that consumption of saturated fats, particularly from red meat and full-fat dairy, may raise circulating estrogen concentrations and contribute to the development of fibroids. In contrast, diets abundant in fiber, fruits, and vegetables appear to exert a protective effect, potentially lowering fibroid risk. Obesity, through increased aromatization and consequent estrogen production, also represents an established risk factor. This narrative review aims to explore the role of nutritional determinants in the onset and progression of uterine fibroids, with a specific focus on the impact of individual nutrients, foods, and dietary patterns on clinical outcomes. Particular emphasis is placed on obesity and macronutrient composition (e.g., high-fat versus high-fiber dietary regimens) as potential modulators of circulating estrogen levels and, consequently, fibroid growth dynamics. Furthermore, the potential of nutritional strategies as complementary therapeutic approaches, capable of integrating established clinical practices, is examined.

## 1. Introduction

Leiomyomas are benign uterine tumors arising from smooth muscle cells and characterized by an extracellular matrix rich in proteoglycans, fibronectin and collagen. Prevalence increases with age, peaking at 40–50 years and affecting women of all ethnicities, with an increased incidence in Black women [1]. While most fibroids are asymptomatic, symptomatic cases can significantly affect quality of life, manifesting as heavy menstrual bleeding (HMB), anemia, and chronic pelvic pain and, in some cases, having a significant impact on women’s fertility. In fact, uterine fibroids are implicated in about 10% of infertility cases and up to 3% as a single factor [2].

Multiple fibroids, particularly in younger women, are thought to result primarily from genetic predisposition, whereas solitary fibroids in older women are more frequently associated with environmental influences leading to exogenous and/or endogenous hyperestrogenism [3].

Fibroid management depends on symptom severity, reproductive goals, and overall health. Options range from pharmacological therapies and surgery (myomectomy, hysterectomy) to minimally invasive approaches such as uterine artery embolization or thermal ablation [4]. Given concerns about fertility, side effects, and recurrence, there is growing interest in complementary and lifestyle-based strategies [2].

As in endometriosis management, where complementary and lifestyle-based therapies are increasingly integrated into multidisciplinary care, similar strategies may also be appropriate for uterine fibromatosis [5]. The frequent coexistence of adenomyosis and fibroids, which share clinical features and pathogenetic pathways, underscores the need for early diagnosis and timely treatment to improve quality of life and limit complications. Fibroid management should therefore integrate pharmacological, surgical, minimally invasive, and complementary approaches, tailored to reproductive goals and overall health [6].

Among these, nutritional factors have emerged as potential modulators of fibroid development and progression. Given the hormone-dependent nature of fibroid growth, dietary patterns capable of influencing estrogen metabolism and systemic inflammation are of particular interest [7]. Epidemiological studies suggest that diets high in fruits, vegetables, vitamins, green tea, and other plant-derived compounds may exert a protective effect, while high consumption of red meat, saturated fats, and alcohol may elevate the risk of fibroid formation [1,2]. Nevertheless, findings remain inconclusive due to methodological variability and population heterogeneity.

In recent years, increasing attention has been devoted to the systemic implications of benign estrogen-dependent gynecological disorders. Similar to endometriosis, which has been reported to co-occur with chronic inflammatory bowel diseases [8] and neurological conditions such as migraine [9], uterine fibroids are now being considered within a broader clinical framework, characterized by extra-gynecological comorbidities and multisystem involvement.

Disparities in fibroid prevalence may also be shaped by socioeconomic and environmental determinants, including unequal access to nutrient-rich foods, higher rates of obesity, vitamin D deficiency, and greater exposure to environmental pollutants [2]. These factors may partly explain ethnic and geographical differences in incidence and severity.

The aim of this narrative review is to explore the current understanding of the relationship between fibroids and nutritional factors. It aspires to identify clinical and epidemiological evidence that may help identify risk and protective factors in the development of leiomyomas. Moreover, this review seeks to explore the possible contribution of nutrition as an adjunctive strategy for managing fibroid-associated symptoms, with a focus on differentiating patients in whom genetic determinants prevail from those more strongly influenced by environmental factors. By identifying gaps in knowledge and highlighting emerging findings, this review also informs future research directions and potential integrative approaches to treatment.

## 2. Materials and Methods

**Figure 1:**
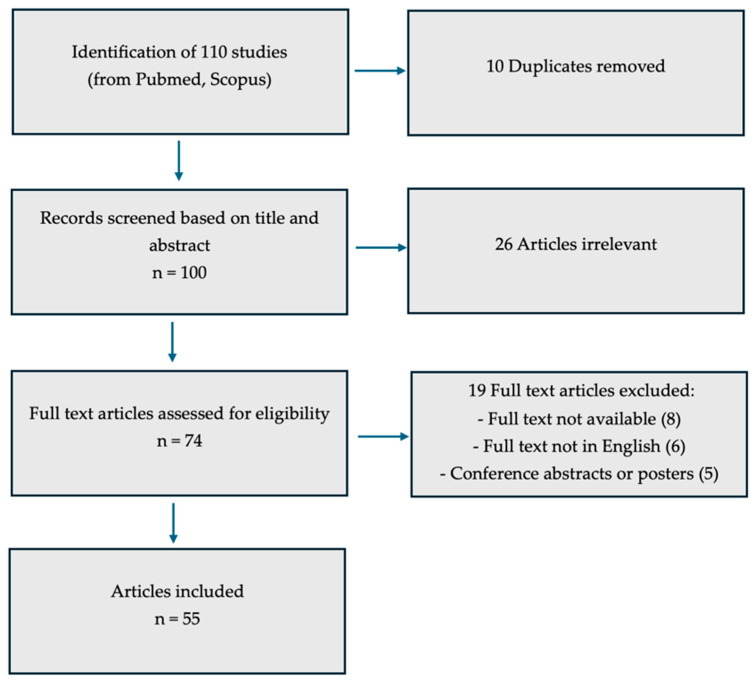
Inclusion criteria diagram.

An electronic search of the MEDLINE database (via PubMed, U.S. National Library of Medicine and Scoups) was performed to identify all English-language publications related to fibromatosis and nutritional factors from its inception through June 2025 (Figure 1). To retrieve relevant studies, we used combinations of specific keywords and Medical Subject Headings (MeSH), including ‘Aliment,’ ‘Diet,’ ‘Fibroids,’ ‘Leiomyomas,’ ‘Myoma,’ ‘Nutrition,’ and ‘Nutritional Factors.’ Eligible sources comprised original investigations such as randomized and non-randomized clinical trials, prospective observational studies, retrospective cohort analyses, and case–control studies, as well as review papers. Studies were included if they explicitly addressed the scope of this narrative review, namely, providing an overview of fibromatosis in relation to nutritional factors.

The study was conducted independently by two researchers (F.G.M. and G.C.), who meticulously reviewed all articles meeting the inclusion criteria. All clinical aspects of the topic were discussed, beginning with the prevalence of disease, pathogenetic theories, diagnostic approach, symptoms, medical and surgical treatment, concluding with the potential role of nutritional factors as a complementary therapy.

Table 1 lists studies investigating the relationship between fibromatosis and nutrition, including the strength of each recommendation (Table 1).

In total, 110 studies were retrieved from PubMed and Scopus. After eliminating 10 duplicates, 100 records were screened by title and abstract, leading to the exclusion of 26 articles considered irrelevant. Of the remaining 74 potentially eligible studies, 19 were removed for the following reasons: eight lacked full-text availability, six were not published in English, and five were conference abstracts or posters. Consequently, 55 studies were ultimately included in this review (Figure 1).

## 3. Epidemiology

It is estimated that more than 70% of women develop uterine fibroids by the time of menopause. Among women of reproductive age, these lesions can be clinically detected in approximately one quarter, and about 25% of affected individuals experience symptoms of sufficient severity to require therapeutic intervention [62].

The true prevalence of fibroids has likely been underestimated in epidemiologic research, as most studies have focused on symptomatic women, overlooking the large number of asymptomatic cases or those who do not report their symptoms [63]. Reported prevalence rates vary considerably—from 4.5% to 68.6%—depending on the type of study, diagnostic methods used, and the ethnic composition of the study population [64].

Data from U.S. studies indicate that by the age of 50, fibroids are detectable by ultrasound in over 80% of women of African descent and in nearly 70% of Caucasian women [65]. However, data on prevalence in other racial and ethnic groups—such as Asian and Hispanic women—are scarce, potentially contributing to further underestimation [63].

The high prevalence of fibroids has major implications for healthcare systems worldwide. In the U.S., the combined annual direct and indirect economic burden of fibroids has been estimated at nearly $34.4 billion [66].

Marked ethnic differences exist in both prevalence and clinical presentation [67]. Women of African ancestry show a higher propensity for fibroid development than Caucasian and Asian women, often manifesting at an earlier age and with a more substantial tumor burden, both in number and size [63]. Additional established risk factors include obesity, nulliparity, hypertension, late menopause, early menarche, and a positive family history [67].

Other factors associated with increased risk are vitamin D deficiency [10,11], alterations in the reproductive tract microbiome [12], and exposure to endocrine-disrupting chemicals such as organophosphate esters and plasticizers [13,68]. Tobacco smoking and alcohol consumption have also been implicated [14,15], and the coexistence of multiple risk factors appears to further increase the likelihood of fibroid development and growth [3].

Oral contraceptive use has been linked to a lower risk of fibroid development and a reduction in associated clinical symptoms [64,69].

In conclusion, although numerous studies have examined the prevalence, risk factors, and protective factors for uterine fibroids, interpretation is often complicated by underdiagnosis. This is largely due to disparities in access to diagnostic tools across different socioeconomic and ethnic groups, as well as the frequent absence of symptoms, which can prevent women from seeking even routine medical evaluation.

## 4. Pathogenesis

Over the past decade and a half, the integration of cutting-edge genomic approaches—most notably high-throughput sequencing—has transformed the molecular understanding of uterine fibroids. These advances have uncovered recurrent, and often mutually exclusive, genetic alterations that drive tumor initiation and growth. The most common alterations are somatic mutations in the Xq13 gene, which encodes MED12, a component of the RNA polymerase II (Pol II) Mediator complex. Such mutations are detected in approximately 45% to 90% of cases, with prevalence varying significantly by patient ethnicity [70,71].

Although less common, other pathogenic alterations have been documented, including HMGA2 overexpression, disruption of the COL4A5–COL4A6 locus, and biallelic loss of FH, the gene encoding fumarate hydratase, a key enzyme of the tricarboxylic acid (TCA) cycle [3,72].

Recurrent chromosomal deletions and rearrangements affecting 6p21, 7q22, 22q, and 1p have also been identified in affected individuals. These lesions typically co-occur with other molecular alterations, indicating that they are more likely secondary drivers within their evolutionary landscape rather than primary initiating events [73,74,75].

In addition to point mutations and structural rearrangements, uterine fibroids frequently exhibit copy-number variations (CNVs), which are present in approximately 40–50% of cases. The most recurrent abnormalities involve rearrangements at 12q14–15 targeting *HMGA2*, gains of chromosome 12, deletions of 7q22–q31, and alterations in 6p21 involving *HMGA1* [76,77,78]. These events often correlate with specific molecular subtypes, as *HMGA2*-driven fibroids usually harbor cytogenetically visible CNVs, whereas *MED12*-mutated tumors tend to lack large chromosomal changes. Recent single-cell and spatial transcriptomic studies have further refined this landscape, showing that leiomyomas are composed of heterogeneous smooth muscle and fibroblast populations, with subtype-specific distributions—*MED12*-mutant fibroids containing balanced SMC and fibroblast proportions, while *HMGA2*-rearranged fibroids are strongly SMC-dominant [79,80]. Stromal fibroblast clusters display transcriptomic programs reminiscent of cancer-associated fibroblasts (CAFs), with high extracellular matrix (ECM) gene expression, suggesting that paracrine crosstalk between mutant SMCs and fibroblasts contributes to fibroid growth and stiffness [80].

The delineation of these distinct driver events has enabled the molecular subclassification of uterine fibroids into at least four genetically defined subtypes, each associated with specific pathogenic pathways and biomarker signatures [81,82,83].

### 4.1. MED12

MED12 mutations have been identified in uterine fibroids among women from diverse ethnic backgrounds—including North American, European, African, Asian, and Middle Eastern populations—highlighting its role as a major driver in leiomyoma pathogenesis [84,85]. A recent meta-analysis, however, reported a significantly higher prevalence of MED12 mutations in women of African ancestry compared with other ethnicities [86]. In most cases, MED12 mutations in fibroids are missense variants located within exon 1 or exon 2, while a smaller subset consists of in-frame deletions or insertions [81,84,87]. Mutations in exon 2 are by far the most common, whereas exon 1 alterations represent a much smaller fraction of the pathogenic variants identified in uterine leiomyomas [3]. Notably, most exon 2 missense mutations cluster at codons 36, 43, and 44, underlining the critical role of these evolutionarily conserved amino acid residues [3]. The notion that MED12 mutations act as true oncogenic drivers is supported not only by their high frequency but also by experimental evidence. For example, targeted expression of a mutant MED12 allele (c.131G>A; p.G44D) in the uterine mesenchyme of mice was sufficient to induce leiomyoma formation, providing direct genetic proof of causality [88]. Integrating molecular findings with clinical data, several studies have shown that MED12 mutations are more often associated with numerous but relatively small fibroids and with tumors of the conventional histological subtype [84,89,90,91]. Although the exact molecular mechanisms remain unclear, pathogenic MED12 mutations are thought to contribute to uterine fibroid formation through disruption of RNA polymerase II–dependent gene expression.

The Mediator complex is a large multiprotein assembly that functions as an essential intermediary between sequence-specific transcription factors and Pol II [87]. It integrates regulatory inputs from both activators and repressors, ultimately reshaping gene expression programs that control diverse physiological processes, including cell growth, homeostasis, development, and differentiation. Structurally, Mediator contains a 26-subunit core that binds tightly to Pol II, forming the so-called holoenzyme. Four additional subunits—MED12, MED13, Cyclin C (CycC), and CDK8 (or its paralog CDK19)—constitute a “kinase module” that associates with the core in a context-dependent manner [87]. This kinase module serves as a primary conduit for signaling through Mediator, and MED12-dependent activation of CDK8 is essential for transmitting nuclear signals from multiple oncogenic pathways with which MED12 is biochemically and genetically linked [87]. Within this module, MED12 directly interacts with CDK8, inducing a conformational rearrangement and stabilizing its activation loop (T-loop) in a manner that depends on amino acid residues recurrently mutated in uterine fibroids [92]. These findings indicate that MED12 driver mutations associated with fibroids may induce structural changes in the T-loop, consequently compromising CDK8 kinase activity [92]. Indeed, pathogenic exon 2 mutations in MED12 have been shown to reduce CDK8/19 kinase activity both in vitro and in patients with clinically significant leiomyomas [71,81,93]. Together, these findings identify a recurring molecular defect in MED12-mutated fibroids, implicating the loss of Mediator-associated CDK8/19 kinase activity in their pathogenesis. Mechanistically, mediator kinase activity participates in numerous cellular processes, from controlling transcription factor stability and Pol II function to modulating chromatin architecture and functional states [87,94,95]. Since MED12 mutations are connected to several signaling pathways directly implicated in fibroid biology—including Wnt/β-catenin, AKT/mTOR, progesterone receptor, focal adhesion, extracellular matrix, angiogenesis, and HIF1α signaling [82,96,97,98], understanding the specific role of CDK8 in MED12-dependent regulation of these cascades—and the extent to which altered Mediator kinase activity contributes to their dysregulation—remains a key area for future research.

Beyond functional studies of the Mediator kinase module, several transcriptomic analyses have directly compared MED12-mutant and MED12-wild-type leiomyomas. These investigations consistently demonstrate that MED12-mutant fibroids harbor distinct gene-expression programs characterized by dysregulation of extracellular matrix organization, Wnt/β-catenin signaling, growth factor pathways, and cell-cycle regulators [82,99,100]. Although inter-tumoral heterogeneity exists, recurrent commonalities—such as upregulation of collagen and fibronectin gene families, altered enhancer activity, and changes in hormone receptor signaling—emerge across different cohorts [101,102]. Collectively, these findings reinforce the concept that MED12 mutations reshape the transcriptional landscape of leiomyomas and provide mechanistic clues to their initiation and growth.

### 4.2. HMGA2

The high mobility group A (HMGA) family comprises the related non-histone chromosomal proteins HMGA1 and HMGA2, which influence transcription by reshaping chromatin structure. These proteins bind to AT-rich sequences within enhancers or promoters, engaging the minor groove of DNA and inducing conformational changes in chromatin structure. Among the most frequent genetic abnormalities in uterine fibroids, occurring in approximately 8–10% of cases, is a translocation between chromosomes 12 and 14 that disrupts a putative regulatory sequence upstream of the HMGA2 gene [103,104]. In fibroids harboring 12q15 rearrangements, HMGA2 expression is significantly higher than in normal myometrial tissue [105]. Fibroids harboring HMGA2 alterations exhibit marked overexpression of the proto-oncogene pleomorphic adenoma gene 1 (PLAG1), indicating that HMGA2 may contribute to fibroid development, at least partially, via activation of PLAG1 [82].

### 4.3. FH

Mutations in the fumarate hydratase (FH) gene, located on chromosome 1, have been identified in uterine fibroids [106,107]. Hereditary leiomyomatosis and renal cell carcinoma (HLRCC) syndrome is caused by heterozygous germline mutations in the FH gene [108]. FH deficiency, accounting for up to 1.6% of uterine fibroids, is associated with a distinct transcriptional profile characterized by strong upregulation of glycolysis-related genes [109] and activation of nuclear factor erythroid 2–related factor 2 (NRF2) target genes [82].

### 4.4. COL4A5/COL4A6

Deletions involving COL4A5 and COL4A6 represent a rare molecular subtype of uterine fibroids, accounting for approximately 2% of cases [3]. Recently, a small set of mutually exclusive driver mutations has been identified, including germline variants in the SRCAP complex components YEATS4 and ZNHIT1. Fibroids with these mutations show impaired incorporation of the histone variant H2A.Z [110]. Although the precise mechanisms underlying genomic instability in uterine fibroids are not yet fully understood, impairments in DNA damage response and repair pathways appear to be major contributing factors. In fibroid tissue, numerous DNA repair genes show reduced expression relative to the adjacent normal myometrium from the same patient, indicating that impaired repair mechanisms may contribute to the onset and growth of these benign tumors [3]. Notably, the genes RAD51 and BRCA1, which play central roles in homologous recombination (HR) repair of DNA double-strand breaks (DSBs), exhibit abnormal expression patterns in fibroid tissues [111,112]. Fibroid-derived stem cells also display a global downregulation of proteins involved in DSB repair—particularly within the HR pathway—alongside altered phosphorylation profiles when compared with mesenchymal stem cells from adjacent myometrium. These changes reflect a dysregulated DNA damage response and an increased burden of DNA lesions, as demonstrated by elevated phosphorylation of histone H2A.X at serine 139 (γ-H2AX) in response to DSB formation [113]. Experimental evidence from animal models supports these observations. Mesenchymal cells isolated from adult rats exposed to diethylstilbestrol (DES) during uterine development show reduced DNA end-joining capacity, elevated DNA damage levels, and impaired DSB repair relative to age-matched controls. These findings indicate that early-life exposure to endocrine-disrupting chemicals may compromise DNA repair capacity in mesenchymal cells, promoting the accumulation of mutations that could contribute to fibroid development later in life [114]. Within the broader context of fibroid pathogenesis, other DNA repair pathways—such as nucleotide excision repair (NER) and base excision repair (BER) mechanisms responsible for removing oxidative DNA lesions—should be explored in future investigations [115]. Epigenetic processes—including DNA methylation, histone modifications, non-coding RNAs, and heterochromatin remodeling—are increasingly recognized as contributors to uterine fibroid pathogenesis [3]. Some studies have reported aberrant DNA methylation and demethylation patterns in fibroid cells [116]. MicroRNAs play a pivotal role in the epigenetic regulation of fibroid development. Significant alterations in the expression of regulatory microRNAs have been documented, particularly within the let-7, miR-21, miR-93, miR-106b, miR-29, and miR-200 families [117,118].

Epigenetic dysregulation in fibroids can activate key intracellular signaling pathways, notably Wnt/β-catenin and Wnt/MAPK [119].

Historically, uterine fibroids have been classified as estrogen-dependent tumors [120]. In fibroid biology, estrogen signaling represents a central molecular pathway that operates through genomic and non-genomic mechanisms, activating signaling cascades like Ras-Raf-MEK-MAPK and PI3K-PIP3-Akt-mTOR. Multiple alterations in estrogen receptors (ERs) and downstream signaling components have been linked to fibroid development [121]. As others gynecology diseases of reproductive age [122], beyond its proliferative effects, estrogen has been shown to upregulate progesterone receptor expression [123], a mechanism also observed within fibroid cells themselves [124]. In vitro, combined treatment with estradiol and progesterone enhances fibroid cell proliferation [125]. Likewise, in an in vivo xenograft model, steroid hormones—including estradiol and progesterone—were found to be essential for tumor growth and maintenance [126].

Overall, the pathogenesis of uterine fibroids is a multifactorial process that remains incompletely understood and requires further research, also with the aim of developing new therapeutic strategies.

**Figure 2:**
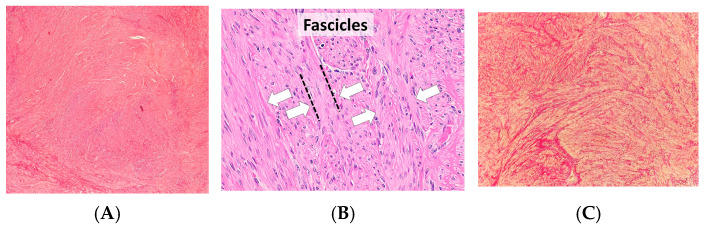
(**A**) Classic leiomyoma. Well-circumscribed nodule composed of intersecting smooth-muscle fascicles; bland spindle cells with elongated nuclei and inconspicuous nucleoli; minimal atypia and low mitotic activity. (**B**) Leiomyoma showing fascicular growth. The white arrows indicate the fascicles of smooth-muscle cells, arranged in parallel and intersecting bundles, highlighting the typical whorled architecture of the tumor; no coagulative tumor cell necrosis is observed. (**C**) Smooth-muscle bundles separated by collagen; special stain emphasizes extracellular matrix content typical of some fibroids [127].

To complement the molecular and genetic insights discussed, representative histological images are provided (Figure 2). These illustrate the typical fascicular arrangement of smooth muscle cells and the abundant extracellular matrix deposition, offering a spatial perspective of the cellular composition. 

## 5. Types of Patients

In line with the pathogenic mechanisms outlined above, two main clinical profiles can be distinguished. Younger patients frequently present with multiple uterine fibroids, a condition in which genetic predisposition appears to be the predominant determinant. In contrast, older women more commonly develop solitary fibroids, in which environmental influences—particularly those leading to exogenous and/or endogenous hyperestrogenism—may exert a decisive role. From a clinical management perspective, these distinctions are relevant, as they suggest that therapeutic strategies may need to be tailored according to the underlying etiological profile [128]. In both scenarios, nutritional interventions represent a promising complementary approach. While diet cannot replace pharmacological or surgical treatments, it may modulate hormonal balance, reduce symptom burden, and possibly attenuate disease progression [16]. The degree of benefit is likely to vary depending on whether genetic or environmental factors predominate, but incorporating nutrition into a broader, multidisciplinary management strategy could provide additional advantages in improving quality of life and overall outcomes for patients with uterine fibroids [1,16].

## 6. Diagnosis

The diagnosis of uterine fibromatosis is established through a comprehensive medical history, physical examination, gynecological evaluation, and pelvic ultrasound imaging. A detailed medical history may also reveal risk factors, such as age, family history, or reproductive history. On physical examination, a healthcare provider may detect an enlarged, irregularly shaped uterus, often indicative of fibroids. These clinical signs, while suggestive, are not definitive, and imaging is essential for accurate diagnosis [129,130].

Ultrasound (US) serves as the first-line imaging modality due to its widespread availability, low cost, and minimal contraindications. Ultrasound provides high sensitivity and specificity in the diagnosis of uterine fibromatosis. Transvaginal ultrasound (TVUS) is considered the most accurate approach for pelvic evaluation and proves particularly useful in obese patients, those with significant bowel gas, or incomplete bladder filling [131]. Conversely, the transabdominal (TAS) approach is more advantageous in cases of significantly enlarged uteri that extend beyond the pelvic cavity, as it enables better visualization of the uterine fundus [132].

On ultrasound, fibroids usually present as well-defined, homogeneous hypoechoic masses compared with the adjacent myometrium, often accompanied by a posterior acoustic shadow. Areas of necrosis appear anechoic, while calcifications present as hyperechoic foci; both features may be visualized within the fibroid lesions [133].

Ultrasound also enables the use of advanced techniques such as Color Doppler (CD), Power Doppler (PD), and three-dimensional (3D) imaging. Doppler modalities are essential for evaluating the vascularization of uterine structures. A defining feature of uterine fibroids is the presence of an intensely vascularized peripheral rim together with a central region characterized by sluggish capillary flow. The assessment of vascular patterns is crucial for differentiating fibroids from other uterine pathologies, such as endometrial polyps, which typically present with a pedunculated vascular pattern and a central feeding vessel [134].

Differential diagnosis with adenomyosis can also be achieved through Doppler imaging. In adenomyotic lesions, the vascularization is characterized by penetrating vessels that produce a speckled vascular flow pattern. Additional sonographic features suggestive of adenomyosis include subendometrial anechoic cystic spaces, globular uterine enlargement, subendometrial echogenic linear striations, uterine wall thickening, heterogeneous myometrial echotexture, and thickening of the junctional zone. Overall, these diagnostic features facilitate the differentiation between adenomyosis and uterine fibroids [135].

The 3D ultrasound technique allows for improved spatial assessment and provides more precise localization of fibroid lesions [136].

Sonohysterography enhances the specificity of ultrasound in evaluating the uterine cavity and assessing its potential involvement, particularly in the case of submucosal lesions [137].

Ultrasound features that may raise suspicion for uterine sarcoma include a large solid mass (e.g., diameter greater than 8 cm), heterogeneous echogenicity, the presence of cystic or necrotic areas, and atypical vascularization—such as irregular or absent blood flow in necrotic regions—differing from the typical peripheral vascular pattern seen in fibroids. When such features are observed, further diagnostic evaluation is warranted to exclude malignancy [138]. MRI is considered the second-line imaging modality for evaluating lesions with uncertain cellularity, such as suspected leiomyosarcomas. It has demonstrated high diagnostic accuracy, with reported sensitivity of 90% and specificity of 96%. 

Another imaging technique with potential utility in this context is Positron Emission Tomography (PET), which, although not routinely used, has shown promise in the assessment of suspicious uterine masses [139].

**Figure 3:**
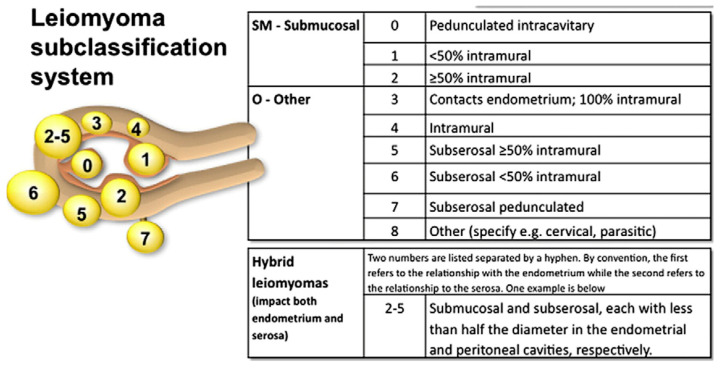
FIGO subclassification system [140].

The current classification is based on the anatomical relationship of the myomatous lesion to the uterine wall and follows the FIGO system, which includes types 0 to 8 (Figure 3). FIGO type 0 refers to entirely intracavitary fibroids that are pedunculated and attached to the endometrium by a narrow stalk. Types 1 and 2 are submucosal fibroids: type 1 lesions have less than 50% intramural extension, while type 2 lesions have 50% or more of their volume within the myometrium. Type 3 fibroids are localized within the myometrium while abutting the endometrial lining. Type 4 fibroids are entirely intramural, with no contact with either the endometrium or the serosa. Types 5 and 6 are subserosal fibroids with an intramural component: type 5 lesions have 50% or more of their volume within the myometrium, whereas in type 6 this proportion is less than 50%. FIGO type 7 refers to pedunculated subserosal fibroids. Lastly, FIGO type 8 includes fibroids located in other positions, such as cervical, parasitic, or those not involving the myometrium, like broad ligament fibroids [140].

## 7. Treatment

Therapeutic options for uterine fibroids comprise medical, interventional radiologic, and surgical strategies. Current guidelines recommend initiating treatment with medical or minimally invasive approaches prior to considering surgical intervention [141]. Medical therapy for fibroids may pursue different goals: symptom-oriented treatment targeting the predominant complaint (e.g., heavy menstrual bleeding), therapies that act simultaneously on symptoms and fibroid volume, and complementary approaches that may synergize with standard treatments to both alleviate symptoms and reduce fibroid size. In cases where pharmacologic or minimally invasive measures prove inadequate, surgical options become necessary.

The choice of procedure must take into account reproductive age and the patient’s desire for future pregnancy. For women seeking fertility preservation, myomectomy is generally the first-line surgical option [142]. Alternative uterine-sparing options include magnetic resonance-guided focused ultrasound surgery (MRgFUS), which achieves thermal ablation of accessible fibroids under MRI guidance, and ultrasound-guided transcervical radiofrequency ablation (RFA), a minimally invasive technique associated with significant long-term symptom relief and fibroid shrinkage, with high patient satisfaction and low complication rates [143,144,145]. Uterine artery embolization (UAE) represents another effective minimally invasive strategy, inducing ischemic necrosis of fibroid tissue while sparing the surrounding myometrium. UAE has demonstrated clinical improvement in over 85% of cases and comparable outcomes to myomectomy in terms of quality-of-life improvement, although evidence regarding fertility and live birth rates remains limited [146,147,148]. Hysteroscopic myomectomy remains the preferred minimally invasive technique for submucosal fibroids (FIGO 0–2), particularly when fertility is impaired by cavity distortion or endometrial inflammation [149]. However, its role is limited in cases involving intramural or subserosal fibroids or concomitant adenomyosis, and evidence regarding improved live birth rates compared with other surgical approaches is inconclusive [146]. Finally, hysterectomy represents the definitive treatment for uterine fibroids, especially in women who no longer desire fertility. It eliminates recurrence and simultaneously treats coexisting pathologies such as adenomyosis or endometrial hyperplasia. Despite its efficacy in ensuring long-term symptom control and sustained quality-of-life improvement, hysterectomy carries surgical risks and should be considered only after thorough counseling and evaluation of patient-specific factors [150,151,152]. Alongside these approaches, non-surgical strategies, including radiologic interventions and complementary nutritional therapies, remain important in tailoring individualized patient care.

### 7.1. Symptomatic Therapy

COCs, composed of estrogen and progestin, are frequently employed to normalize menstrual cyclicity and to reduce heavy menstrual bleeding associated with fibroids. While they are effective in controlling symptoms, they do not reduce fibroid volume. They may be appropriate for women with mild symptoms who are not actively pursuing pregnancy [153]. Levonorgestrel-Releasing Intrauterine System (LNG-IUS) is an intrauterine device which releases levonorgestrel locally within the uterine cavity, reducing endometrial proliferation and leading to significant reductions in menstrual blood loss. However, its efficacy is limited in cases with large or submucosal fibroids, which may increase the risk of device expulsion [154]. The use of an antifibrinolytic agent, such as Tranexamic Acid, can be useful to control heavy menstrual bleeding. It does not affect fibroid size but can significantly improve quality of life in patients with excessive bleeding. It is taken only during menstruation [155].

### 7.2. Pharmacological Therapies Targeting Fibroid Volume

#### 7.2.1. GnRH Agonists

Gonadotropin-releasing hormone (GnRH) agonists—such as triptorelin, leuprorelin, and goserelin—suppress the hypothalamic-pituitary axis, leading to decreased estrogen and progesterone production. They are used short-term to reduce fibroid size and manage abnormal uterine bleeding, mainly as preoperative therapy or a bridge to menopause or alternative treatments [156]. Their administration requires parenteral delivery or the use of long-acting implantable formulations due to their peptide structure. Treatment with GnRH agonists improves hemoglobin concentrations and reduces surgical blood loss, though the therapeutic effect is short-lived, with fibroids generally recurring within 3 to 9 months following withdrawal. Adverse effects include hypogonadism-related symptoms, altered lipid metabolism, and reduced bone mineral density, which can be mitigated with add-back therapy (ABT). Treatment is usually limited to 6–12 months, depending on the ABT regimen used [150].

#### 7.2.2. GnRH Antagonists

GnRH antagonists—such as Elagolix, Linzagolix, and Relugolix—are orally administered non-peptide molecules that exert their effects by directly inhibiting GnRH receptors at the pituitary level. Their oral bioavailability represents a key advantage over traditional peptide-based GnRH analogs. By suppressing the hypothalamic-pituitary-gonadal axis, these agents reversibly inhibit the secretion of follicle-stimulating hormone (FSH) and luteinizing hormone (LH), resulting in decreased ovarian production of estradiol and progesterone. This hypoestrogenic state contributes to therapeutic efficacy in hormone-dependent conditions like uterine fibroids but is also associated with adverse effects such as hot flushes, headaches, hypertension, and bone mineral density (BMD) loss. To mitigate these effects—particularly bone loss—add-back therapy with low-dose estrogen and progestin is recommended. Clinical trials have demonstrated the efficacy of GnRH antagonists in significantly reducing menstrual blood loss (MBL), improving anemia, and reducing fibroid volume. These agents temporarily suppress ovulation, with symptoms often recurring upon discontinuation [157]. Elagolix, in both monotherapy and combination regimens, has been shown to reduce MBL and increase amenorrhea rates compared to placebo. Hemoglobin levels improved, reflecting effective management of anemia [158]. Patients receiving Elagolix with add-back therapy also reported greater symptom relief and improved quality of life, as measured by the Uterine Fibroid Symptom and Quality of Life (UFS-QoL) questionnaire [159]. Similar results were observed with the administration of relugolix, which demonstrated a dose-dependent reduction in menstrual blood loss. Treatment with relugolix also led to improvements in hemoglobin levels and symptom severity, alongside reductions in total uterine and fibroid volume—particularly with the higher 40 mg dose [160]. The use of Relugolix in combination with estradiol and norethindrone acetate has been evaluated in preoperative settings for women with abnormal uterine bleeding due to fibroids. A three-month course resulted in a ≥2 g/dL hemoglobin increase in 61.3% of patients, along with reductions in fibroid size and overall uterine volume. These changes enabled surgical plan modifications—such as converting laparotomies to minimally invasive procedures—and, in some cases, postponement of surgery due to clinical improvement, allowing continued medical management [161]. The LIBERTY study further assessed the long-term efficacy and safety of Relugolix combination therapy (40 mg Relugolix, 1 mg estradiol, 0.5 mg norethindrone acetate) in women with heavy menstrual bleeding due to fibroids. At week 76, 78.4% of patients maintained MBL < 80 mL, compared to 15.1% in the placebo group (*p* < 0.0001). Efficacy persisted, with 69.8% maintaining control at week 104 versus 11.8% with placebo (*p* < 0.0001). Amenorrhea was achieved or sustained in 57.4% at week 76 and 58.3% at week 104, significantly outperforming placebo. Importantly, the combined regimen demonstrated good tolerability, with no additional safety signals observed throughout the second year of therapy. One of the main concerns with GnRH-targeting therapies—BMD reduction—was significantly mitigated by add-back therapy. Over a 24-month period, mean BMD loss was <2% at the lumbar spine, with minimal changes at the femoral neck and total hip, remaining within clinically acceptable limits. No cases of clinical osteoporosis or fractures were reported [162]. Overall, Relugolix combination therapy offers an effective and well-tolerated long-term treatment option for premenopausal women with uterine fibroids. By balancing symptom control with bone health preservation, it provides a valuable non-surgical alternative, particularly for patients who are not suitable surgical candidates or who wish to preserve their uterus [163].

#### 7.2.3. Danazol and Gestrinone

Danazol and Gestrinone are synthetic steroids with anti-gonadotropic activity. They reduce fibroid-related symptoms, particularly bleeding, by inducing a hypoestrogenic, hyperandrogenic state. It was also shown a potential in reducing fibroid volume and uterine size, though evidence remains scarce and of low quality. Therapeutic application of these agents is constrained by androgenic side effects—including acne, hirsutism, and weight gain—as well as by symptoms related to hypoestrogenism [164].

#### 7.2.4. SPRMs

Selective progesterone receptor modulators (SPRMs), such as ulipristal acetate, mifepristone, vilaprisan, and asoprisnil, act through mixed agonist-antagonist activity on progesterone receptors, reducing fibroid proliferation and promoting apoptosis. These agents are effective in decreasing fibroid volume and controlling abnormal uterine bleeding [165]. Ulipristal acetate yielded favorable results in the PEARL I and II clinical trials [166]. However, its long-term use has been limited due to safety concerns, notably cases of severe hepatotoxicity [167]. As a result, its indication is now restricted to perimenopausal women who are not candidates for surgery or have experienced surgical failure. During treatment, regular liver function monitoring and patient awareness of hepatic risk symptoms are strongly recommended [168].

#### 7.2.5. Statins

Statins are primarily used for lipid regulation; they are being investigated for anti-proliferative and pro-apoptotic effects on fibroid cells. Though preclinical results are promising, more clinical trials are needed before they can be routinely recommended [169].

### 7.3. Complementary Therapy

Complementary therapies are defined as non–first-line interventions used alongside conventional medical or surgical treatments with the purpose of enhancing therapeutic outcomes, alleviating symptoms, reducing adverse effects, and improving patients’ overall quality of life. In contrast to alternative therapies, which are employed instead of standard treatments, complementary therapies act synergistically with evidence-based care [164,170,171]. Their use is common among women with uterine fibroids, either combined with conventional management or, in selected cases, as stand-alone approaches when symptoms are mild or fertility preservation is a priority. Jacoby et al. reported that up to one-third of women with fibroids resort to complementary strategies, reflecting a strong interest in non-invasive and holistic options [172]. The most frequently described approaches include dietary modifications, phytotherapy, and nutritional supplementation. Mind–body interventions, particularly acupuncture, have received growing attention. Acupuncture has been shown to alleviate pelvic pain, reduce heavy menstrual bleeding, and improve fatigue and emotional distress associated with fibroids. By modulating neuroendocrine pathways and pain perception, it offers symptomatic relief and contributes to improved quality of life [171,173]. Other lifestyle interventions, such as weight control and regular physical activity, may indirectly influence fibroid progression by reducing estrogen levels through decreased adipose aromatization, while also improving cardiovascular and metabolic health [164]. Although the strength of evidence varies and much of the available data is observational, the widespread use of these approaches underscores their relevance in clinical practice. Integrating complementary strategies—ranging from acupuncture and mind–body practices to lifestyle and selected nutraceutical interventions—into a multidisciplinary care plan may provide synergistic benefits, particularly for women reluctant to undergo surgery or those seeking to optimize reproductive and overall health outcomes [171,172].

## 8. Nutritional Factor

Nutrition plays a central role in numerous gynecological diseases, particularly those related to estrogen [17]. Uterine fibroids appear to be influenced by multiple determinants, including hormonal balance, genetic predisposition and environmental factors. Excessive exposure to estrogen is one of the main mechanisms hypothesized for the development of these benign neoplasms [3]. In this context, nutritional factors emerge as potentially modulating risk factors: foods rich in saturated fats, such as those found in red meat and whole dairy products, seem to promote increased estrogen levels, encouraging the growth of these formations. Conversely, diets high in fiber, fruit and vegetables are associated with a protective effect, probably due to their ability to modulate hormone metabolism [1].

### 8.1. Nutritional Factor and Pathogenesis

Most investigations into the relationship between nutrition and uterine fibroids have primarily focused on specific nutritional factors—such as vegetables, fruits, carotenoids, soy-derived products, and vitamin D—and their possible effects on fibroid prevalence. Although interest has been increasing, the molecular mechanisms that explain these associations are still only partly clarified. Consequently, long-term, non-invasive therapeutic strategies for uterine fibroids are still limited [3]. Furthermore, the extent to which dietary habits influence the onset and progression of these lesions remains largely uncertain.

#### 8.1.1. Vegetables and Fruits

A consistent body of evidence suggests an inverse association between a diet rich in fruits and vegetables and the risk of developing uterine fibroids. Phytochemicals—bioactive compounds found in fruits, vegetables, cereals, legumes, nuts, and seeds—include flavonoids, carotenoids, and polyphenols. These molecules are known to modulate processes such as cell proliferation, inflammation, fibrosis, apoptosis, and angiogenesis [18]. In an in vitro setting, leiomyoma cells were exposed for 48 h to methanolic extracts derived from two phytochemical-rich strawberry cultivars, Romina (R) and Alba (A), as well as to an anthocyanin-enriched extract from Romina (RA). Treatment with both R and RA markedly reduced the expression of extracellular matrix proteins, including collagen, fibronectin, activin A, and versican. Among the tested preparations, RA exhibited the strongest pro-apoptotic effect in fibroid cells [19].

Although these findings remain preliminary, fruit, vegetable, and fiber intake may already offer indirect clinical benefits by supporting weight control, improving insulin sensitivity, and reducing systemic inflammation—all mechanisms strongly implicated in fibroid pathophysiology. Well-designed clinical trials are needed to determine whether these dietary strategies can directly modify fibroid incidence, growth, or symptom severity.

#### 8.1.2. Dairy Products

Milk-derived foods are a source of minerals and vitamins such as calcium, magnesium, and vitamin D, which may contribute to reducing tumorigenesis and inflammation [20]. In a large prospective cohort study including over 81,000 premenopausal women and more than 8000 incident fibroid cases, Orta et al. found that women in the highest quintile of dietary calcium intake had a modestly reduced fibroid risk (HR = 0.92; 95% CI: 0.86–0.99), and higher yogurt consumption (≥2 servings/day) was also associated with lower risk (HR = 0.76; 95% CI: 0.55–1.04); however, the overall relationship between total dairy intake and fibroid incidence remained weak and inconclusive [20].

#### 8.1.3. Soy

Soy is a major dietary source of isoflavones, plant-derived non-steroidal phenolic compounds which are among the most estrogenic compounds known. Isoflavones belong to the phytoestrogen class and share structural similarities with 17β-estradiol [21]. It has been proposed that high consumption of soy-rich foods may affect the endocrine system, potentially influencing fibroid development and growth. However, current evidence is still limited [22]. In a meta-analysis, Qin et al. assessed the influence of soy-based products on fibroid prevalence, including studies on soy intake in both childhood and adulthood. Childhood consumption was associated with a 35% increase in risk, which rose to 92% when only adult women were considered [1,23].

#### 8.1.4. Green Tea Extracts

Green tea, derived from Camellia sinensis, contains high levels of catechins—such as epigallocatechin gallate (EGCG), epigallocatechin, and epicatechin gallate—with EGCG being the most abundant and biologically active. EGCG exhibits anti-angiogenic and anti-proliferative properties and has been investigated as a potential therapeutic agent for several conditions, including those affecting the female reproductive tract [1]. Experimental studies, both in vitro and in vivo, have shown that EGCG induces apoptosis in fibroid cells and animal models, leading to a reduction in fibroid number and size [24]. EGCG has been shown to downregulate cyclin D1—an overexpressed cell cycle regulator in fibroid cells—and to inhibit the production of extracellular matrix components such as collagen and fibronectin [18]. EGCG has been shown to downregulate cyclin D1—an overexpressed cell cycle regulator in fibroid cells—and to inhibit the production of extracellular matrix components such as collagen and fibronectin [165]. In a randomized pilot trial including 39 women with symptomatic fibroids, Roshdy et al. reported that daily administration of 800 mg of green tea extract (45% EGCG) for 4 months significantly reduced fibroid volume (−32.6% vs. +24.3% with placebo) and improved symptom severity scores (−32.4% vs. −4.7%), as well as menstrual blood loss and quality of life [25]. Similarly, in an observational study, Biro et al. found that women taking EGCG-enriched green tea extract capsules (390 mg/day) reported improved quality of life, although no significant changes in fibroid size or number were detected [26].

#### 8.1.5. Selenium

Selenium is a trace element with well-recognized antioxidant activity and the ability to regulate heat shock protein 70 (Hsp70) expression. Its potential role in uterine fibroid biology has been explored in an experimental study on Japanese quails, a species in which fibroids can occur spontaneously. In the selenium-supplemented group, fibroid size was significantly smaller than in controls, although the number of tumors did not differ between groups [27]. While these findings suggest a possible protective effect, well-designed human studies are still lacking.

#### 8.1.6. Curcumin

Curcumin, a polyphenolic compound derived from Curcuma longa, has demonstrated the capacity to inhibit proliferation across multiple tumor cell lines. Its precise mechanisms remain incompletely elucidated, though they likely involve modulation of signaling pathways and extracellular matrix remodeling. In a murine xenograft model using human leiomyoma tissue, curcumin administration resulted in marked reductions in tumor growth and extracellular matrix protein synthesis [169]. Complementary in vitro work on rat-derived uterine leiomyoma cells confirmed its antiproliferative action [28]. Although promising, these data are preliminary and require confirmation in clinical settings.

#### 8.1.7. Vitamin D

Vitamin D has received the greatest attention among micronutrients in relation to uterine fibroids. Beyond its established role in calcium–phosphate homeostasis, it participates in cell cycle control, differentiation, and immune modulation [11]. Endogenously produced through sunlight exposure and obtainable from dietary sources, vitamin D has been shown to suppress fibroid cell proliferation, whereas deficiency may foster inflammatory processes in the myometrium [20]. Its biological activity is mediated through the nuclear vitamin D receptor (VDR) and activation of tyrosine kinase–dependent signaling. Immunohistochemical analyses have revealed reduced VDR expression within fibroid tissue compared with adjacent myometrium, and in perilesional areas relative to unaffected muscle [29]. Fibroids have also been found to exhibit increased expression of CYP24A1, which encodes 24-hydroxylase—the enzyme that metabolizes vitamin D3—indicating a potential local deficiency in functional vitamin D [30]. Clinical evidence supports a potential protective role of vitamin D: in a prospective ultrasound cohort of 1.610 women with ≥1 follow-up scan, serum 25-hydroxyvitamin D levels ≥ 20 ng/mL (vs. <20 ng/mL) were associated with an estimated 9.7% reduction in fibroid growth (95% CI: −17.3% to −1.3%); moreover, levels ≥ 30 ng/mL (vs. <30 ng/mL) corresponded to a 22% lower incidence of fibroids (adjusted HR = 0.78; 95% CI: 0.47–1.30), as well as a 32% increase in fibroid loss (adjusted risk ratio = 1.32; 95% CI: 0.95–1.83). These results lend support to the hypothesis that higher vitamin D concentrations inhibit fibroid development and promote regression, though the small number of women with serum levels ≥ 30 ng/mL limits precision [31]. A 12-month supplementation trial demonstrated reductions in fibroid volume and decreased surgical intervention rates [32]. Moreover, in a pilot study, a three-month regimen combining vitamin D (50 μg), epigallocatechin gallate (EGCG, 300 mg), and vitamin B6 (10 mg) resulted in a significant decrease in fibroid size [33]. Given the widespread prevalence of vitamin D deficiency worldwide [34], alongside mounting evidence of its systemic health benefits, vitamin D may be a valuable adjunct in strategies aimed at slowing fibroid development and progression.

Although reduced sunlight exposure at higher latitudes could plausibly contribute to vitamin D deficiency, current evidence derives mainly from serum 25(OH)D measurements rather than cross-country comparisons, and latitude-specific data on fibroid incidence remain limited [29,30,31,32,33].

Interventional trials are needed to confirm these associations and to define optimal dosing strategies.

### 8.2. Nutritional Factor and Symptoms

The clinical presentation of uterine fibroids is influenced by the size, number, and anatomical location of the lesions, with common manifestations including heavy menstrual bleeding, secondary anemia, dysmenorrhea, chronic pelvic pain, symptoms related to pelvic organ compression, and, in some cases, dyspareunia and infertility. Emerging evidence indicates that nutritional factors may substantially affect not only the risk of fibroid onset but also their progression and the severity of associated symptoms [35]. A diet high in saturated fat and low in fibre has been associated with increased levels of circulating estrogen, due to both increased endogenous synthesis and decreased intestinal excretion of steroid hormones [36]. This hyperestrogenic state may promote fibroid growth and exacerbate HMB, thereby increasing the risk of iron-deficiency anemia. In cases of large fibroids or those located in the submucosa, excessive proliferation can be aggravated by a chronic low-grade inflammatory state, promoted by unbalanced eating habits (e.g., excessive consumption of simple sugars, red meat and ultra-processed foods), worsening pelvic pain and gastrointestinal symptoms (bloating, flatulence, constipation) [37,38]. Compression symptoms, such as pollakiuria, urinary urgency or persistent constipation, can also be indirectly affected by body weight and metabolic status. In fact, visceral obesity not only promotes the peripheral aromatization of androgens into estrogens but also mechanically contributes to increasing pressure on the pelvic organs, amplifying urinary and intestinal disorders [39]. Numerous observational studies have reported an association between obesity and increased fibroid volume, increased risk of recurrence after treatment, and worsening of the clinical picture [23,39,40]. In this context, weight loss through a low-calorie, balanced diet can lead to significant clinical improvement, even in the absence of surgical treatment.

In this scenario, nutritional factors may play a dual role: as preventive agents on the one hand and as potential supportive tools in therapy on the other. Some studies also suggest that adequate intake of nutrients such as omega-3 fatty acids, along with dietary approaches that limit glycaemic load, may reduce dysmenorrhea and chronic pelvic pain, probably through the modulation of prostaglandins and local inflammatory mediators [41,42,43]. Finally, regular fibre intake improves intestinal transit and may reduce pelvic discomfort caused by faecal stasis in cases of rectal compression from posterior fibroids [44]. Overall, nutritional factors emerge as significant and modifiable elements in the control of symptoms in uterine fibroids. A nutrition-based approach, integrated into a multidisciplinary treatment plan, represents a non-invasive, sustainable and customisable strategy for improving the quality of life of affected patients.

### 8.3. Nutritional Factor and Therapy

**Figure 4:**
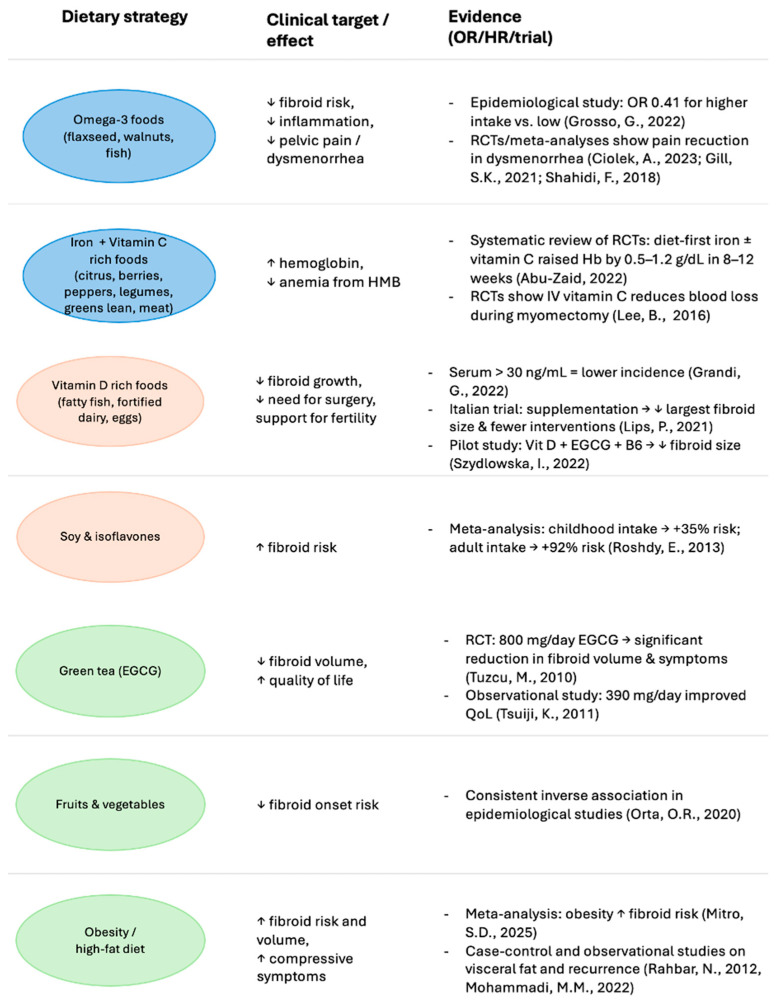
Evidence-based dietary strategies in the management of uterine fibroids: symptom-oriented approach. Color coding indicates the primary symptom domain targeted: **Blue (Metrorrhagia/anemia)**—iron- and vitamin C–rich foods, together with omega-3 sources, may counteract anemia and reduce bleeding intensity; **Green (Pelvic pain/symptoms)**—anti-inflammatory foods such as green tea, fruits, and vegetables, and avoidance of high-fat diets, may alleviate uterine pain, reduce inflammation, and improve quality of life; **Orange (Infertility)**—vitamin D–rich foods may support fertility and reproductive health, and reduce the need for surgical interventions [20,25,27,28,33,34,35,41,42,43,44,45,50,56,60,61].

Although several dietary classes and bioactive compounds have been investigated for their role in the pathogenesis and incidence of uterine fibromatosis, current scientific evidence does not yet support their use as established therapeutic agents. However, the following compounds represent those with the most promising therapeutic potential based on available preclinical and clinical data. A summary of the available evidence on dietary and nutritional strategies for uterine fibroids is reported in Figure 4.

#### 8.3.1. Omega-3 Polyunsaturated Fatty Acids (PUFAS)

Omega-3 polyunsaturated fatty acids (PUFAs) are well known for their anti-inflammatory and membrane-stabilizing properties, which have been widely studied in various chronic inflammatory conditions such as cardiovascular disease and rheumatoid arthritis [45,46,47]. These compounds are also emerging as potentially relevant in the context of benign gynecological conditions, including uterine fibromatosis.

Although current data are somewhat conflicting, some epidemiological studies suggest a protective association between omega-3 intake and fibroid development. In particular, women with higher dietary omega-3 fatty acid intake showed a markedly lower risk of uterine fibroids (OR = 0.41), indicating a potential risk reduction. Clinically, these findings support the hypothesis that nutritional modulation through omega-3 supplementation or diet may contribute to prevention and symptom management in affected women. At the mechanistic level, omega-3 fatty acids have been shown to remodel plasma membrane architecture and downregulate genes involved in mechanotransduction and lipid storage in leiomyoma cells, changes that may underlie their observed clinical effects [48].

Despite these promising insights, results remain inconsistent, and further studies are needed to clarify the dose–response relationship, the influence of different omega-3 types (EPA, DHA), and the effect of dietary versus supplemental intake in fibroid pathophysiology. Nevertheless, their well-established safety profile and systemic anti-inflammatory action make omega-3 fatty acids an intriguing area for future research in the non-hormonal management of uterine fibromatosis.

#### 8.3.2. Polyphenols, Such as Resveratrol and Curcumin

Among the dietary bioactives investigated for their impact on uterine fibroids, polyphenols—especially resveratrol and curcumin—have shown promising antiproliferative and pro-apoptotic properties. Resveratrol, a stilbene commonly found in grapes and berries, has demonstrated the ability to suppress leiomyoma cell proliferation and promote apoptosis, with animal studies supporting its role in modulating key pathways of fibroid growth and survival, including cell cycle arrest and oxidative stress regulation [34,49]. Similarly, curcumin has exhibited significant antifibrotic and pro-apoptotic activity, reducing fibronectin expression and inhibiting ERK/NF-κB signaling, mechanisms implicated in leiomyoma pathogenesis [7]. Although clinical data remains scarce, these compounds are considered safe and well tolerated in humans, and preliminary findings suggest they may complement conventional medical treatments, improve symptom control and potentially reducing recurrence risk. Their incorporation into nutritional or supplementation strategies thus represents a feasible adjunctive approach that warrants further evaluation in randomized clinical trials [50,51]. Overall, while further clinical trials are needed to define effective dosages and long-term effects, the incorporation of specific polyphenols into dietary strategies represents a concrete and promising perspective in the supportive care of uterine fibromatosis.

#### 8.3.3. Vitamin D

Growing evidence underscores the pivotal role of vitamin D in regulating uterine fibroid growth, supporting its potential utility as both a preventive measure and an adjuvant therapy, especially in women with confirmed deficiency [7].

Biologically, vitamin D is involved in several mechanisms that counteract fibroid development. It inhibits cell proliferation, promotes apoptosis, regulates inflammation, and modulates the expression of genes related to the extracellular matrix (ECM)—all key factors in the pathogenesis of fibromatosis. Moreover, stable vitamin D analogs are currently under investigation and show promising therapeutic potential [52].

An Italian clinical trial involving 108 women with ‘small burden’ uterine fibroids and concomitant hypovitaminosis D showed a negative correlation at baseline between serum 25-OH-D and fibroid volume (r = −0.18, *p* = 0.01). In that study, supplementation with vitamin D for 12 months was associated with a lower rate of progression needing medical or surgical intervention (13.2% vs. 30.9% in controls, *p* = 0.05), and fibroid volumes remained stable in the supplemented group while increasing modestly in the non-supplemented group. These data lend tangible clinical support to the concept that restoring adequate vitamin D levels may help curb fibroid progression [32].

On a molecular level, vitamin D exerts antifibrotic effects by downregulating the expression of transforming growth factor beta 3 (TGF-β3) and extracellular matrix proteins. It also regulates the activity of matrix metalloproteinases (MMPs), key enzymes responsible for ECM remodeling. In vitro studies have shown that vitamin D inhibits the growth of uterine fibroid cells by downregulating proliferating cell nuclear antigen (PCNA) and cyclin-dependent kinase 1 (CDK1), as well as suppressing the expression and activity of catechol-O-methyltransferase (COMT), an enzyme involved in estrogen metabolism. Vitamin D has also been reported to reduce the expression of estrogen and progesterone receptors, exerting antiestrogenic and antiprogesterone effects. Furthermore, it may inhibit the activation of the Wnt/β-catenin signaling pathway, which is involved in cellular proliferation, and participate in DNA repair mechanisms, thus reducing genomic instability associated with fibroid pathogenesis [53]. Overall, these findings support the use of vitamin D—whether through nutritional supplementation, controlled sun exposure, or targeted pharmacological supplementation—as a complementary strategy in the management of uterine fibromatosis, particularly in women with vitamin D deficiency. Given its safety, low cost, and the high prevalence of vitamin D deficiency worldwide, supplementation may represent a particularly feasible adjunct in clinical practice, although larger randomized trials are warranted to confirm efficacy and establish optimal regimens.

#### 8.3.4. Obesity and Insulin Resistance

Robust epidemiological evidence identifies obesity as a significant risk factor for the development and growth of uterine fibroids. Adipose tissue contributes to increased peripheral estrogen production via aromatase activity, creating a hyperestrogenic environment conducive to fibroid growth. Additionally, obesity is often associated with insulin resistance and chronic low-grade inflammation, both of which may promote fibrogenesis [3,54]. Nutritional and lifestyle interventions aimed at weight reduction and metabolic improvement may therefore exert indirect but clinically relevant therapeutic effects, not only reducing fibroid risk but also alleviating symptom severity. Caloric restriction, low-glycemic index dietary patterns, higher fiber intake, and regular physical activity have all been shown to improve insulin sensitivity and decrease systemic inflammation [55]. Emerging data suggest that addressing insulin resistance may also reduce fibroid volume or growth rate, although randomized controlled trials are still lacking. In the context of conservative management, lifestyle modification—particularly structured dietary and exercise interventions—should be considered a cornerstone of non-pharmacological therapy for women with fibroids and concurrent metabolic dysregulation [56].

#### 8.3.5. Iron Intake and Anemia Management

Heavy menstrual bleeding secondary to fibroids is a leading cause of iron-deficiency anemia (IDA), which worsens fatigue, cognitive function and surgical risk. Targeted nutritional strategies therefore sits beside pharmacologic therapy as a key pillar of supportive care. Heme iron from lean red meat, fish and liver is absorbed more efficiently than non-heme iron from pulses and leafy greens. Co-ingestion of vitamin-C-rich foods (citrus, berries, bell peppers) enhances non-heme absorption, whereas phytates, polyphenols and calcium inhibit it; spacing tea/coffee or high-calcium foods ≥ 2 h from iron-rich meals can improve uptake [57]. Importantly, a 2022 systematic review of 14 randomized controlled trials in women with IDA found that diet-first interventions—such as iron-fortified foods or iron-plus-vitamin-C meals—raised hemoglobin by approximately 0.5–1.2 g/dL over 8–12 weeks, with fewer gastrointestinal side effects compared to oral tablets. Notably, the review did not identify a standardized dosage of iron or vitamin C, as the included randomized trials employed different formulations and amounts delivered through fortified foods or enriched meals, rather than fixed supplement regimens [58]. These data underscore that structured nutritional interventions (e.g., iron-fortified foods or iron-plus-vitamin-C meals) can represent a clinically relevant and well-tolerated strategy to correct anemia in women with fibroids, particularly for those who cannot tolerate supplements or are awaiting surgical treatment.

### 8.4. Nutritional Factor and Surgery

Nutritional strategies are increasingly being explored as supportive measures in the perioperative management of uterine fibromatosis. Evidence suggests that specific dietary interventions may help optimize surgical outcomes and recovery [1,164]. For instance, preliminary evidence suggests that vitamin C supplementation during abdominal myomectomy may help reduce intraoperative blood loss, shorten surgical duration, and minimize hospital stay. However, results in the setting of laparoscopic procedures remain inconsistent and require further confirmation [59,60,61]. Beyond single nutrients, integrated lifestyle programs combining dietary counseling with physical activity, such as the Lifestyle Intervention for Fibroid Elimination (LIFE) study, have demonstrated improvements in symptom severity, quality of life, and vitamin D levels over 12 months, highlighting the potential of structured nutritional approaches as adjuncts to surgical care [16]. In clinical practice, emphasis is often placed on a diet rich in protein, iron, vitamins, and adequate hydration, both before and after surgery, to support tissue healing, counteract anemia, and reduce postoperative complications. Although current evidence remains limited and largely observational, these findings suggest that nutritional therapy may play a valuable complementary role in surgical management, improving not only perioperative outcomes but also long-term health status and overall patient well-being [164].

## 9. Comparison with the Literature and Study Limitations

The results discussed in this review are in line with the most recent scientific literature, which highlights the growing role of diet in modulating the hormonal and inflammatory factors involved in the genesis and progression of uterine fibroids. Findings from both observational and experimental studies indicate that diets high in saturated fats and refined sugars are linked to an elevated risk of developing fibroids and worsening related symptoms, whereas dietary patterns rich in fiber, antioxidants, and polyunsaturated fatty acids appear to exert a protective effect. However, most of the available evidence is based on observational studies, which often have heterogeneous designs and significant variability in diagnostic criteria, dietary assessment methods and follow-up times. Moreover, disentangling the specific contribution of diet from potential confounders—including lifestyle, hormonal milieu, and genetic predisposition—represents a methodological challenge consistently observed across many of the studies reviewed. A strength of this review is the integrated and multidimensional approach adopted, which combines clinical evidence with biochemical and pathophysiological considerations. The nutritional strategies proposed are not limited to the management of physical symptoms but are part of a broader context of promoting women’s general health and psychophysical well-being, offering complementary and sustainable support to traditional therapies.

## 10. Future Perspective

Although current evidence highlights some nutritional factors as clinically relevant—such as vitamin D, obesity-related metabolic dysregulation, and iron intake—many other promising areas remain underexplored. Future investigations should focus on how nutraceuticals and bioactive compounds can modulate key pathogenetic mechanisms of fibroids, including extracellular matrix remodeling, hormone receptor signaling, oxidative stress, angiogenesis, and chronic inflammation [119]. Epigenetic regulation also deserves attention: alterations in 5-hydroxymethylcytosine patterns and dysregulated microRNAs have been implicated in leiomyoma growth, suggesting potential nutritional and nutraceutical interactions [116]. Similarly, links between DNA damage/repair mechanisms and fibroid development point to novel research directions [111].

At the genetic level, MED12 mutations and fumarate hydratase deficiency remain central in fibroid biology, and future studies could explore whether specific dietary or supplemental factors influence these pathways. Environmental exposures, including alcohol consumption and endocrine-disrupting chemicals, may further interact with nutritional determinants to modify risk [13,15].

Another important gap concerns the potential differential impact of nutritional factors on distinct fibroid subtypes (submucosal, intramural, subserosal). Most available studies evaluate overall risk or fibroid burden without stratification, and whether location-specific responses to dietary or nutraceutical interventions exist remains to be clarified. Addressing this question could help tailor nutritional strategies more precisely to clinical presentation [1,2,3].

Overall, integrating nutritional interventions with advances in molecular and genetic research may help identify new therapeutic targets. Well-designed trials should assess whether bioactive compounds such as polyphenols, omega-3 fatty acids, selenium, or soy-derived components can modulate these mechanisms. Such efforts could bridge molecular insights with clinical practice, paving the way for personalized, non-hormonal strategies in fibroid management.

## 11. Conclusions

Uterine fibroids represent one of the most common gynecological conditions, particularly among women of reproductive age. The integration of complementary therapies offers the potential to alleviate symptoms while acting synergistically with conventional medical or surgical interventions, thereby allowing for reduced dosages of standard treatments and minimizing associated side effects without compromising efficacy. In particular, targeted dietary strategies may not only improve fibroid-related symptoms but also mitigate the risk of metabolic comorbidities such as diabetes, hypertension, and dyslipidemia. Such approaches could provide substantial benefits by enhancing women’s quality of life and, at the same time, reducing healthcare costs, ultimately contributing to improved outcomes at both the individual and population level.

Current evidence supports a link between nutritional factors and fibroid development and progression. Clinical and preclinical studies consistently point to vitamin D status, obesity-related metabolic dysregulation, and iron intake in the context of heavy menstrual bleeding as clinically relevant elements. Correcting vitamin D deficiency, addressing obesity and insulin resistance, and optimizing iron intake therefore represent feasible and safe adjunctive strategies in fibroid management.

From a scientific perspective, several bioactive compounds (e.g., EGCG, resveratrol, curcumin, omega-3 fatty acids) show promising mechanisms of action but still require clinical validation. Their integration into comprehensive management plans offers promising opportunities for translational research.

## Figures and Tables

**Table 1:** Evidence on Diet, Nutrition and Gynecologic Health. ↓: worse ↑: improve.

Author/Title	Study Type	Nutritional Therapy	Mechanism	Improvement in Symptoms/Disease	Level of Recommendation
Krzyżanowski J et al., Nutrients 2023 [1]	Review	Diet quality, vit D, dairy	Hormonal; anti-inflammatory	Yes	B
Afrin S et al., Nutrients 2021 [2]	Review of clinical studies	Various (vit D, omega-3)	Endocrine; inflammation	Yes	B
Tinelli A et al., IJERPH 2021 [7]	Narrative review	General diet	Estrogen metabolism; oxidative stress	Yes	C
Mohammadi R et al., RB&E 2020 [10]	SR/MA	Vitamin D	Anti-proliferative	Yes	A
Ciebiera M et al., IJMS 2018 [11]	Review	Vitamin D	VDR signaling	Yes	B
Baker JM et al., Front Immunol 2018 [12]	Review	Microbiome (indirect)	Immune modulation	Not	C
Bariani MV et al., Curr Opin Endocrinol 2020 [13]	Review	Contaminant reduction	Endocrine disruption	Not	C
Wong JY et al., Fertil Steril 2016 [14]	Prospective	Dietary factors	Hormonal/metabolic	Yes	C
Takala H et al., Curr Mol Med 2020 [15]	Review	Alcohol reduction	Estrogen; oxidative stress	Yes	C
Bellon M et al., Reprod Sci 2025 [16]	Pilot interventional	Diet + supplements	Weight; anti-inflammatory	Yes	B
Martire FG et al., J Clin Med 2025 [17]	Review	Anti-inflammatory diet; omega-3	Inflammation; immune	Yes	B
Islam MS et al., Pharmacol Rep 2017 [18]	Review	Phytochemicals	Anti-proliferative	Yes	B
Giampieri F et al., (in vitro) [19]	In vitro	Strawberry phytochemicals	Anti-proliferative	Not	C
Orta OR et al., Hum Reprod 2020 [20]	Prospective cohort	Dairy	Calcium/vit D; IGF	Yes	B
Křížová L et al., Molecules 2019 [21]	Review	Isoflavones	SERM; antioxidant	Not	C
Belobrajdic DP et al., Nutrients 2023 [22]	Review	Soy foods	Microbiota; anti-inflammatory	Not	C
Qin H et al., JECH 2021 [23]	SR/MA	Weight management	Adiposity-estrogen	Yes	A
Hazimeh D et al., Nutrients 2023 [24]	Review	Green tea/EGCG	Anti-proliferative	Yes	B
Roshdy E et al., IJWH 2013 [25]	Pilot RCT	EGCG	Anti-proliferative	Yes	B
Biro R et al., Arch Gynecol Obstet 2021 [26]	Observational	EGCG	Anti-proliferative	Yes	C
Tuzcu M et al., Nutr Cancer [27]	Animal study	Selenium	Antioxidant	Not	C
Tsuiji K et al., Gynecol Endocrinol 2011 [28]	In vitro	Curcumin	Anti-proliferative	Not	C
Markowska A et al., Nutrients 2022 [29]	Observational (IHC)	Vitamin D	VDR involvement	Not	C
Othman ER et al., EJOGRB [30]	Observational (tissue)	Vitamin D	Dysregulated enzymes	Not	C
Harmon QE et al., Fertil Steril 2022 [31]	Prospective cohort	Vitamin D status	Endocrine modulation	Yes	B
Ciavattini A et al., Medicine 2016 [32]	Observational	Vitamin D	Anti-proliferative	Yes	C
Grandi G et al., Gynecol Endocrinol 2022 [33]	Pilot interventional	Vit D + EGCG	Synergy anti-prolif	Yes	B
Lips P et al., JBMR Plus 2021 [34]	Review/epidemiology	Vitamin D status	Endocrine/immune	Not	C
Szydłowska I et al., Nutrients 2022 [35]	Review	Vitamins & natural compounds	Anti-fibrotic; hormonal	Yes	C
Wiggs AG et al., Front Endocrinol 2021 [36]	Review	Diet + exercise	↓ Estrogens; inflammation	Yes	C
Vafaei S et al., Nutrients 2024 [37]	Perspective/Review	Lifestyle & diet	Weight; environment	Not	C
Hess JM et al., Adv Nutr 2021 [38]	Review	Dairy	Anti-inflammatory	Not	C
Sun K et al., Exp Ther Med 2019 [39]	Case–control	Weight control (indirect)	Adiposity; estrogen	Not	C
Sefah N et al., Front Pharmacol 2023 [40]	Review	Diet/lifestyle	Socio-environmental	Not	C
Rahbar N et al., IJGO 2012 [41]	RCT	Omega-3	Prostaglandins/resolvins	Yes	B
Mohammadi MM et al., EJCP 2022 [42]	SR/MA	Omega-3	Anti-inflammatory	Yes	A
Ciołek A et al., Nutrients 2023 [43]	Survey	Dietary patterns	Behavioral	Not	C
Gill SK et al., Nat Rev Gastro Hepatol 2021 [44]	Review	Dietary fibre	Microbiota; SCFA	Yes	C
Shahidi F & Ambigaipalan P, ARFST 2018 [45]	Review	Omega-3	Anti-inflammatory	Yes	C
Bäck M & Hansson GK, FASEB J 2019 [46]	Review	Omega-3	Pro-resolving mediators	Yes	C
Raad T et al., Nutrients 2021 [47]	Systematic review	Anti-inflammatory diet/omega-3	↓ Inflammation	Yes	B
Harris HR et al., Fertil Steril 2020 [48]	Prospective cohort + biomarkers	Dietary fat profile	Lipid-hormone interplay	Yes	B
Novakovic R et al., Life 2022 [49]	Review	Resveratrol	SIRT1; antioxidant	Not	C
Grosso G et al., Nutrients 2022 [50]	Review	Anti-inflammatory nutrients	Cytokines/adipokines	Yes	C
Alasalvar C et al., Nutrients 2023 [51]	Review	Dried fruits	Microbiota; antioxidant	Not	C
Laganà AS et al., 2017 [52]	Review	Vitamin D	Reproductive endocrine	Yes	C
Ali M et al., Acta Pharmacol Sin 2019 [53]	In vitro/ex vivo	Vitamin D3	↓ DNA damage	Not	C
Kn S et al., Pathol Res Pract 2025 [54]	Observational (pathology)	Lipid management (indirect)	Lipid accumulation	Not	C
Sun Y et al., BMJ Open 2023 [55]	Cross-sectional	Sedentary reduction (lifestyle)	Adiposity; hormones	Not	C
Mitro SD et al., JCEM [56]	Cohort analysis	Glycemic/dietary control	Insulin/IGF; inflammation	Not	C
Petraglia F & Dolmans MM, Fertil Steril 2022 [57]	Editorial	Iron repletion	Hematologic restoration	Yes	C
Skolmowska D et al., Nutrients 2022 [58]	SR of RCTs	Dietary iron strategies	↑ Iron status	Yes	A
Pourmatroud E et al., Arch Gynecol Obstet 2012 [59]	RCT	IV Vitamin C	↓ blood loss	Yes	B
Abu-Zaid A et al., (SR & MA of RCTs) [60]	SR/MA	IV Vitamin C	↓ perioperative blood loss	Yes	A
Lee B et al., EJOGRB [61]	RCT (double-blind)	IV Vitamin C	Antioxidant; hemostatic	Yes	B

## Data Availability

The data that support the findings of this study are available from the corresponding author, E.P, upon reasonable request.
